# Research and Development of Microphysiological Systems in Japan Supported by the AMED-MPS Project

**DOI:** 10.3389/ftox.2021.657765

**Published:** 2021-04-29

**Authors:** Seiichi Ishida

**Affiliations:** ^1^Division of Applied Life Science, Graduate School of Engineering, Sojo University, Kumamoto, Japan; ^2^Biological Safety Research Center, National Institute of Health Sciences, Kawasaki, Japan

**Keywords:** microphyisiological systems, AMED-MPS project, long-term exposure, cholestasis, fibrosis, liver zonation, liver-MPS

## Abstract

Microphysiological systems (MPS) have been actively developed as a new technology for *in vitro* toxicity testing platforms in recent years. MPS are culture techniques for the reconstruction of the specific functions of human organs or tissues in a limited space to create miniaturized human test systems. MPS have great promise as next-generation *in vitro* toxicity assessment systems. Here, I will review the current status of MPS and discuss the requirements that must be met in order for MPS to be implemented in the field of drug discovery, presenting the example of an *in vitro* cell assay system for drug-induced liver injury, which is the research subject in our laboratory. Projects aimed at the development of MPS were implemented early in Europe and the United States, and the AMED-MPS project was launched in Japan in 2017. The AMED-MPS project involves industry, government, and academia. Researchers in the field of drug discovery in the pharmaceutical industry also participate in the project. Based on the discussions made in the project, I will introduce the requirements that need to be met by liver-MPS as *in vitro* toxicity test platforms.

## Introduction

The development of a new drug takes a long period of time (~10–15 years) and costs ~300 billion yen. This implies that it will take a long time for effective new drugs to reach patients; even if new drugs are developed, their cost may make it difficult for these patients to receive treatment. One of the factors responsible for this situation is the complexity and number of steps involved in the drug development process, starting with the screening of tens of thousands of compound libraries, followed by numerous evaluation tests, including efficacy evaluation and safety confirmation tests. This step is necessary to ensure the safety of a chemical substance that will be administered to humans for the first time and to guarantee the therapeutic efficacy and safety of the drug once it is approved. A wide range of tests exist to aid in this process. *In vitro* tests are often used in the exploratory screening and initial developmental stages of new drugs, but as development progresses, it enters the phase of non-clinical testing, and animal testing becomes an instrumental method. To confirm drug efficacy using disease models, confirm systemic effects, and evaluate the occurrence of side effects and adverse events, which must take into account the uptake, distribution, metabolism, and excretion of compounds in the body (ADME), we inevitably rely on laboratory animals. However, compared to *in vitro* testing, animal studies require a long testing period and large quantities of test substances, leading to higher development costs and longer development periods. Furthermore, it is known that there are inter- and intra-species variations in drug response, with animal experiments having limitations in terms of extrapolation to humans. To solve these problems, “human-relevant” culture methods are required. In recent years, with the advancement in technologies that induce the differentiation of human-derived stem cells, such as iPS cells, into various organs and tissues, there has been progress in the development of culture methods that resemble the environment of human organs and tissues more closely. In this context, microphysiological systems (MPS), that mimic the microenvironment of organs and tissues by co-culturing multiple cells that constitute organs and tissues in a microspace, and applying mechanical stimuli derived from the expansion and contraction that these cells undergo in the organs and tissues and from the flow of blood and other fluids, have emerged. MPS attempt to overcome the problems encountered with conventional culture methods described above by culturing cells in a microspace (often a closed space) that creates an environment with mechanical stimulation and a concentration gradient of oxygen and nutrients due to perfusion of the culture medium. The introduction of MPS into the drug development phase is expected to permit humanized *in vitro* cell assays that predict human physiology more accurately in situations where species differences are an issue, and to replace time-consuming animal experiments, giving patients earlier access to more effective and safer medications. Therefore, in this review, I would like to discuss the current state of MPS development and the points that should be taken into account to enable their usefulness in drug development, focusing mainly on the liver, which is an important organ for drug safety and toxicity assessment, based on the results of the Japanese project in which I participate.

## Microphysiological Systems

There is still no standard definition for MPS. For example, according to the draft definition proposed by the Food and Drug Administration (FDA), an MPS is defined as “an *in vitro* platform composed of cells, explants derived from tissues/organs, and/or organoid cell formations of human or animal origin in a micro-environment that provides and supports biochemical/electrical/mechanical responses to model a set of specific properties that define organ or tissue function^1^”. Simply put, it is a culture method for the reconstruction of the specific functions of human organs or tissues in a limited space. An important aspect of the FDA's definition seems to be the use of microfabrication techniques to create a microenvironment “to model a set of specific properties that define organ or tissue function.” To achieve this, it is essential to incorporate not only technologies for creating microculture spaces, but also elemental technologies for maintaining the culture environment, processing the culture surface to maintain the cells, and biomimicry by utilizing the flow of culture medium, characteristic of microspaces, such as laminar flow, depending on the purpose.

## Trends in MPS Development

A search on PubMed using the keywords “microphysiological system” showed that “microphysiological system” has been in use since 2013 and has been widely used since 2017. The number of papers increased from 4 in 2013 to 20 in 2017, and increased consistently to 46 in 2020. This may reflect the fact that the development of MPS in Europe and the United States reached new heights during this period. For example, a closed organization called “CAAT Transatlantic Think Tank on Toxicology” that brought together researchers involved in MPS research and development, companies, and regulators from across the Atlantic (Europe and the United States), held its first meeting in 2015 (Marx et al., 2016), with a follow-up meeting in 2019 (Marx et al., 2020). In Europe, the Organ-on-Chip in development (ORCHID) was initiated in 2017^2^, and reports on its activities have been published (Franzen et al., 2019; Mastrangeli et al., 2019a,b). This activity has now been taken over as EUROoCS^3^. In the United States, the National Center for Advancing Translational Sciences (NCATS)^4^ initially took the lead (Low and Tagle, 2017; Tagle, 2019), but a discussion forum called the IQ Microphysiological Systems Affiliate^5^ was established within the IQ Consortium (International Consortium for Innovation and Quality in Pharmaceutical Development) in 2017. The IQ Consortium is a forum where about 40 pharmaceutical and biotech companies collaborate to discuss the contributions of science and technology to patients and regulators^6^, and the IQ MPS Affiliate is a forum where companies in the pharmaceutical industry collaborate with the NIH and FDA to discuss the applications of MPS as *in vitro* cell assay systems in drug discovery. Twenty-one pharmaceutical companies have joined the IQ MPS Affiliate^5^. The discussions that took place within this forum have been summarized and published in several reviews within the last 2 years (Ainslie et al., 2019; Baudy et al., 2019; Fabre et al., 2019; Fowler et al., 2019; Hardwick et al., 2019; Philips et al., 2019). Regulatory authorities, such as the FDA, in collaboration with the Defense Advanced Research Project Agency (DARPA)^7^ or the IQ MPS Affiliate, have been involved since early days and have been active in proposing the definition of MPS, as described above, and conducting evaluation studies using MPS commercially available from CN Bio^8^. The variety of MPS products currently available on the market, especially those packaged with both culture media and peripheral equipment, is gradually increasing.

A typical product available today is the OrganoPlate® from Mimetas (Leiden, Netherlands). The specifications are based on the 384-well plate of the SBS standard, and it is possible to evaluate 40–96 different samples by constructing a relatively simple flow channel at the bottom. A variety of cells, including hepatic cells (Bircsak et al., 2021), can be seeded in the flow path, and Mimetas provides its applications on their website^9^. The culture platform is quite simple, as the medium perfusion system in the flow channel is gravity-induced, generated by continuous tilting of the plate with a seesaw shaker. Another example is the PhysioMimix, commercially available from CN Bio. The development of the PhysioMimix was focused on its application as liver and small intestine microenvironments, and as mentioned earlier, the results of the joint research with the FDA have already been published (Rubiano et al., 2020). Emulate (Boston, MA)^10^ is based in Boston and supplies products based on the organs-on-a-chip technology developed by the Wyss Institute for Biologically Inspired Engineering at Harvard University. The flow path of the culture medium is provided on the top and bottom of the stretchable porous cell culture membrane, enabling the culture of various cell combinations, and the mechanical stress caused by stretching the cell culture membrane makes the cells highly functional. Kits and protocols are already available for the central nerve system, lungs, kidney, liver, and small intestine, and papers have been published for each organ (Jang et al., 2013, 2019; Sances et al., 2018; Kasendra et al., 2020; Nawroth et al., 2020). The ParVivo™ Chip provided by Nortis (Woodinville, WA)^11^ can be configured for a variety of microculturing environments and is also available as a pre-seeded cell chip. In addition, a joint research project with NASA is underway to test kidney function in space. TissUse (Berlin, Gremany)^12^, a company which originated from Technische Universität Berlin, provides HUMIMIC chips and control units for cell culture, biopsies, tissue sections, etc., according to the user's needs. Co-culture of multiple organs is possible, with reports published on skin and liver culture (Tao et al., 2021). InShpero (Schlieren, Switzerland)^13^ offers 3D InSight^TM^ using spheroids. The core system is the Akura™ Flow Microphysiological System, which cultivates pre-formed spheroids in a row of culture pores. The bottoms of a series of culture pores are connected, creating a gravity induced flow of culture medium. Reports have been published on spheroids derived from various organs (Gupta et al., 2021). In addition, although still in barrack-type assembled MPS, MPS are also available from Chip Shop^14^, based in Germany, and Micronit^15^, based in the Netherlands. These are not suitable for use in drug development as users must devise the assembly of the perfusion device and the routing of the flow path; but we believe that they will play an important role in expanding the scope of basic research on the applications of MPS.

## The AMED-MPS Project

**Figure 1:**
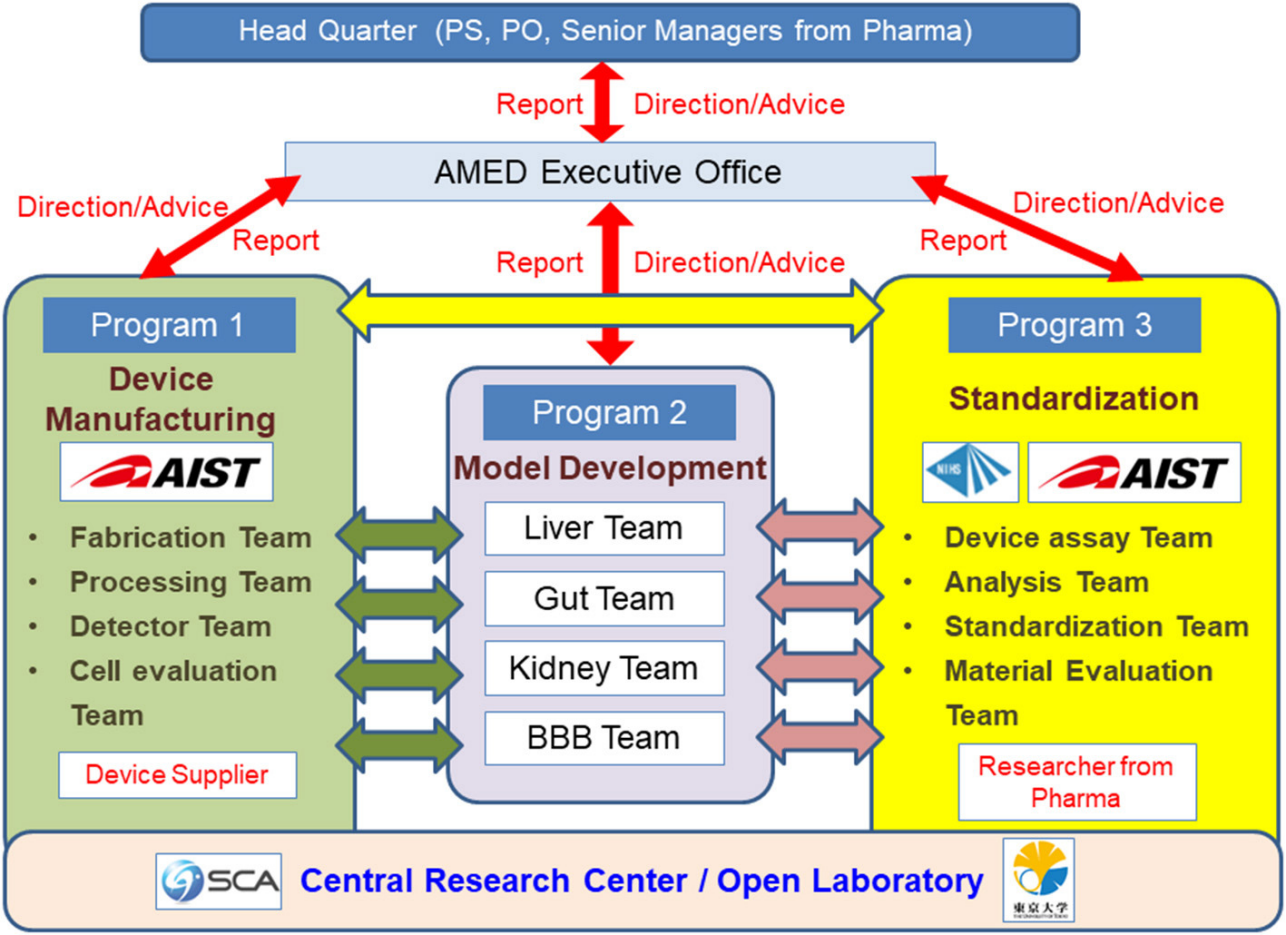
Research and Development Organization of the AMED-MPS Project. Researchers from the National Institute of Advanced Industrial Science and Technology (AIST) and the National Institute of Health Sciences (NIHS) coordinate the manufacturing (Program 1), performance evaluation, and standardization (Program 3) of MPS developed by universities and other academic institutions (Program 3). Device suppliers (Program 1) and pharmaceutical companies (Program 3) equally participate in this project. The target organs/tissues are the liver, small intestines, kidneys, and blood-brain barrier.

In Japan, while the development of MPS in laboratories at universities and other institutions has been effective, the development of commercial MPS has been slow. In response to this, the AMED-MPS project was launched in 2017, led by the Japanese Agency for Medical Research and Development (AMED), with the support of the Ministry of Economy, Trade and Industry. In this project, researchers from the National Institute of Advanced Industrial Science and Technology (AIST) and National Institute of Health Sciences (NIHS) coordinate the manufacturing processes (Program 1), and performance evaluation and standardization (Program 3) of MPS developed by university laboratories (Program 2) (Figure 1). Laboratories that participate in Program 2 are the leading laboratories in MPS development in Japan and are listed in Table 1. In addition, suppliers who produce and commercialize MPS, and pharmaceutical companies that use them in drug development are part of this project, i.e., programs 1 and 3, respectively (Figure 1). The target organs/tissues in this project are the liver, small intestine, kidney, and blood-brain barrier, and the development of MPS is carried out at the Central Research Center in Tsukuba. Moreover, a commercially available MPS was installed in an open laboratory at the University of Tokyo to verify the applications proposed by suppliers and to obtain data by following their proposed protocols. Such data, called “benchmark data,” would be helpful in comparing the performances of commercially available MPS to those of MPS developed under the AMED-MPS project. These laboratories provide a forum for discussion on the performance standards that must be met by the MPS being developed. They also carry out studies to help bring MPS development from lab-scale to the industry, and to develop applications based on those already applied in drug development. It is worth noting that the Pharmaceuticals and Medical Devices Agency (PMDA), which is the regulatory authority for the Japanese pharmaceutical industry, as of 2020, began participating as an observer in the discussions. Four years have passed since the project was initiated, and four types of MPS are already in the product development stage, with the indication of their advantages as MPS.

**Table 1:** List of Laboratories participating Program 2 in AMED-MPS project.

**Research group leader**	**Affiliated University**	**Laboratory URL**
Yasuyuki Sakai	The University of Tokyo	http://orgbiosys.t.u-tokyo.ac.jp/sakai/index.php?lang=en&page=top
Kazuo Takayama	Kyoto University	https://www.cira.kyoto-u.ac.jp/e/research/takayama_summary.html
Tamihide Matsunaga	Nagoya City University	https://www.nagoya-cu.ac.jp/phar/english/index.html
Hiroshi Kimura	Tokai University	http://www.mech.u-tokai.ac.jp/english/theme/index.html
Ryuji Yokokawa	Kyoto University	http://www.ksys.me.kyoto-u.ac.jp/en/
Shoji Takeuchi	The University of Tokyo	http://www.hybrid.t.u-tokyo.ac.jp/en/
Michiya Matsusaki	Osaka University	http://www.chem.eng.osaka-u.ac.jp/~matsusaki-lab/

At the start of the AMED-MPS project, the participating pharmaceutical companies established the items listed in Table 2 as the functions required for the evaluation of drug-induced liver injury, which remains a problem in drug development, using liver-MPS. In the following sections, we will discuss the efforts of Japanese laboratories, including ours, toward the development of assessment systems for long-term exposure, cholestasis, and fibrosis by using hepatocytes, as listed in Table 2.

**Table 2:** Functions required for liver-MPS to evaluate drug-induced liver injury.

➢ Long-term repeated exposure to mimic living organisms • Maintaining pharmacokinetic properties • Bio-mimetic drug exposure kinetics • Toxicity by metabolites • *Biomarkers*: Expression of phase I or phase II enzyme activity Expression of transporter function Induction of CYPs ➢ Coverage of various toxicity mechanisms • Cholestasis • Fibrosis • Immune response • *Biomarkers*: Bile pocket formation Biliary transporter expression and localization Stellate cell activation, collagen expression Non-paranchymal cell function

### Long-Term Exposure

Cryo-preserved human hepatocytes prepared from the human liver are widely used as an *in vitro* system for liver function testing. However, it is difficult to maintain cryo-preserved human hepatocytes for a long period of time using conventional culture methods (Hewitt et al., 2007; Gerets et al., 2012; Heslop et al., 2017); this makes them unsuitable for long-term exposure studies. To resolve this, various improvements have been made to the culture method for human cryo-preserved hepatocytes. Ohkura et al. reported that human cryo-preserved hepatocytes could be cultured in vessels with micropatterning on the cell culture surface to maintain long-term drug metabolism activity (Ohkura et al., 2014). Using the same method, Ogihara et al. showed that it is possible to assess long-term exposure to 11 different drugs (Ogihara et al., 2015). Tetsuka et al. used a 3D-bioprinted liver tissue model, consisting of human primary hepatocytes, stellate cells, and umbilical vein endothelial cells, to successfully reproduce the hepatotoxicity observed with low-dose long-term exposure to acetaminophen *in vitro* (Tetsuka et al., 2020). In addition, media that can maintain human hepatocytes in culture for long periods of time are now commercially available, i.e., Cellartis® Power™ Primary HEP Medium (Takara Bio, Kusatsu, Japan) and HepExtend Supplement (Oriental Yeast Co., Ltd., Tokyo, Japan). These media can maintain the function of cryo-preserved hepatocytes for up to 2–4 weeks, which could not be achieved by conventional culture media. The results of one of our previous studies showed that human cryo-preserved hepatocytes could be cultured in a Cellartis® Power™ Primary HEP Medium for 28 days while maintaining their morphology (Horiuchi et al., manuscript in preparation) and the expression of major drug metabolism-related genes, such as *CYP3A4*. Moreover, long-term culture enhanced the formation of bile canaliculi with bile excretion function (Horiuchi et al., manuscript in preparation, see below for the detail). This is an interesting case that suggests that the choice of culture medium is also important when developing cell culture-using MPS, as it is assumed that long-term culture can be achieved by simply changing the culture media from those used in conventional culture methods.

### Predictive System for Cholestasis

Hepatocytes are not only responsible for the metabolism of nutrients absorbed in the digestive tract, which reach the liver through the portal vein, but also for the production of bile. Some drugs are known to cause cholestasis by inhibiting the function of bile excretion transporters (Susukida et al., 2015, 2016; Takemura et al., 2016). In the clinical trial phase of drug development, elevated AST and bilirubin levels are considered markers of cholestasis, and based on experience, the trial is usually terminated if these increase above a certain level (Hy's law, Robles-Diaz et al., 2014; Dara et al., 2016). Discontinuation of drug development at the clinical trial phase has a significant impact on the development plan, and this is why there is a need for a predictive system for cholestasis that can be used at an early stage of development (Table 2). Conventionally, transporter expression vesicles have been used to evaluate cholestasis. This is partly due to the fact that it is difficult to form sufficient capillary bile ducts in *in vitro* cell culture systems, making it difficult to establish assay systems in cells. However, Takezawa et al. succeeded in efficiently forming capillary bile ducts using an ad-Med Vitrigel (Kanto Chemical, Tokyo, Japan) culture method (Osihkata-Miyazaki and Takezawa, 2016). The Vitrigel, developed by Takezawa et al. is a membrane made of high-density collagen fibers, which gives a highly adhesive culture surface, and also has the property of being permeable to oxygen and small molecules (Takezawa et al., 2007). Taking advantage of this property, in addition to the application of Vitrigel for eye irritancy test (Vitrigel EIT, Yamaguchi et al., 2013), Takezawa et al. established a culture method in which HepG2 cells were cultured on a Vitrigel membrane, with the opposite side of the culture surface placed in the air phase to activate the cells and promote capillary bile duct formation. In addition, our group observed that capillary bile duct-like structures are efficiently formed not only in human cryo-preserved hepatocytes but also in human induced pluripotent stem cell (iPS cell)-derived hepatocytes through long-term culture in the long-term culture medium mentioned in the previous section. Immunohistochemical staining confirmed that the transporters, MRP2, involved in bilirubin excretion, and BSEP, involved in bile excretion, are formed along this capillary bile duct structure. Furthermore, studies using fluorescent model substrates of these transporters showed that MRP2 and BSEP were functional, and their substrate excretion activities were inhibited by their respective inhibitors (Horiuchi et al., manuscript in preparation). These results suggest that the prediction system for cholestasis can be reproduced *in vitro* by developing a culture method.

### *In vitro* Fibrosis Evaluation System

Liver fibrosis is a disease that can lead to cirrhosis and hepatocellular carcinoma, and early treatment is desirable. Viral hepatitis B and C used to be the main causes of the disease; however, most cases are now attributed to alcoholic steatohepatitis and non-alcoholic steatohepatitis (NASH) (Wree et al., 2013; Kostrzewski et al., 2020). Particularly, the prevalence of NASH in Japan is expected to increase in the future as a result of the increased prevalence of lifestyle-related diseases caused by changes in dietary habits, and the development of an *in vitro* evaluation system that is useful for elucidating the etiology and developing therapeutic agents is of utmost importance (Table 2). Liver fibrosis is thought to be caused in part by the activation of stellate cells in the space of Disse (Friedman, 2008). When the liver is damaged, they are activated and become proliferative, producing inflammatory cytokines and collagen that cause fibrosis. To elucidate its pathogenesis and develop therapeutic agents, it is necessary to understand the mechanism of stellate cell activation and to develop screening systems for compounds that inhibit this activation. In addition, it is known that when stellate cells prepared from healthy livers are seeded in normal culture dishes for culture, they are activated, and it is difficult to maintain them in a quiescent state. Shimada et al. reported that LI90 cells, a cell line of stellate cells, change from an activated state to an inactivated state, similar to quiescence, when cultured on Matrigel (Shimada et al., 2010). We believed that the condition of the scaffold in the culture environment was an important stimulus for stellate cell activation, and we investigated the changes induced by culture in the VECELL culture insert (Cosmo Bio, Tokyo, Japan), which was developed by Kodama et al. (Furutani et al., 2008). It is a cell culture scaffold consisting of an expanded polytetrafluoroethylene (ePTFE) mesh coated with collagen type I derived from salmon. Cells placed on VECELL adhere to the mesh surface, where there is no excess scaffold for cells to stretch. Thus, unlike the surface of normal plastic cell culture plates, VECELL allows for the culture of cells under physiological conditions without mechanical stress. When LI90 cells or cryo-preserved stellate cells prepared from human liver were cultured on VECELL culture inserts, they showed spheroid-like morphology. The expression of the activation markers, collagen type I, and smooth muscle actin was significantly decreased, indicating that the cells were in a deactivated state (Horiuchi et al., 2018). Since the stimulation of cells with lipopolysaccharide, which is known to activate stellate cells, suppressed spheroid formation, it was considered to be important for the deactivation of astrocytes. Furthermore, we hypothesized that if we could supply stellate cells as spheroids, we could analyze their activation and deactivation, and provide an evaluation system for compounds that control this process (Shimada et al., 2010; Horiuchi et al., 2018). We attempted to culture the cells in EZSPHERE (AGC Techno Glass, Shizuoka, Japan), that can supply uniform spheroids at once. As a result, we established a culture method that could supply a large number of spheroids with a diameter of ~100 mm. When the prepared spheroids were seeded into normal culture dishes, they developed a fibroblast-like morphology over time, with an increase in the expression of stellate cell activation markers. These results indicate that spheroid culture of stellate cells is an important culture technique, as it supplies stellate cells near quiescence, which is necessary for the evaluation of liver fibrosis (Horiuchi et al., manuscript in preparation).

## Integration of MPS and Culture Technology

In the sections above, we have presented culture techniques for the reconstruction of *in vitro* long-term exposure, cholestasis, and fibrosis, which are required for the evaluation of drug-induced liver injury using MPS. Because MPS are derived from micro-total analysis systems (μTAS) and microelectromechanical systems (MEMS), they tend to be associated with the technology that applies microfabrication technology to culture materials to form micro-culture spaces for the cultivation of cells. However, merely reducing the size of the culture compartment is simply a high-throughput version of the conventional plastic culture dish. As we have seen, to model a set of specific properties that define organ or tissue function, it is important to develop appropriate culture media, culture methods, and culture surfaces (Low et al., 2020). Furthermore, Japan has the infrastructure needed to provide excellent technologies for such culture methods and equipment, such as ad-Med Vitrigel (Takezawa et al., 2007), VECELL culture insert (Furutani et al., 2008), Genocel (Nikke Medical, Osaka, Japan) (Matsuno et al., 2020), Cellbed (Japan Vilene, Tokyo, Japan) (Ikari et al., 2021), and Nano Culture Plate (Oriental Yeast Co., Ltd., Tokyo, Japan) (Eguchi et al., 2018). It is prudent to consider the combination of such culture technology with Japan-made MPS developed under the AMED-MPS project.

## Hepatic Zonation and MPS

**Figure 2:**
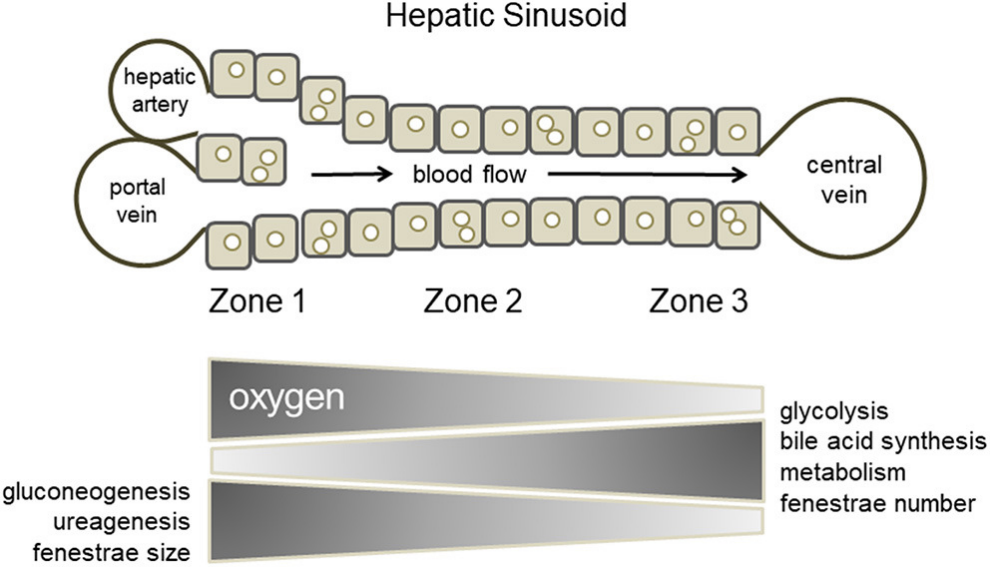
Hepatic zonation. Hepatocytes within the lobule are divided based on differences in their functions between regions (zone 1, zone 2, and zone 3).

**Figure 3:**
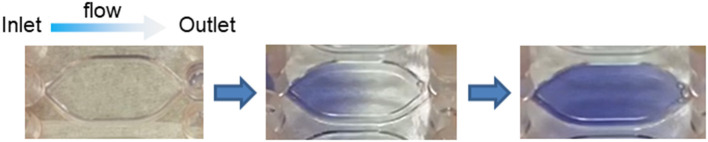
Laminar flow in MPS. Undisturbed flow, called laminar flow, as observed in a commercially available MPS chip. The blue liquid was introduced into an MPS chip filled with a colorless transparent liquid. The colorless liquid was replaced by the blue liquid over time without disturbing the flow.

When considering drug-induced liver injury, one characteristic feature is the phenomenon of site-specific hepatocellular damage in liver lobules. For example, phosphorus poisoning produces necrosis around the portal vein of the liver lobule, while intoxication with acetaminophen and other drugs produces necrosis around the central vein. As shown by the site-specificity of the toxicity expression, it is known that hepatocytes in the hepatic sinusoidal structure between the area near the portal vein and hepatic artery, where blood flows in, and the area around the central vein, which is the exit of the sinusoid, respond differently to chemical substances. In addition, in terms of cellular functions, glycogenesis and urea synthesis are predominant in the portal region, and energy is produced by oxidative metabolism, whereas, bile acid synthesis and metabolism are predominant in the central venous area, and energy is produced by glycolysis. The division of hepatocytes within the lobule based on the differences in hepatic function between regions is called “hepatic zonation” (Figure 2) (Gebhardt and Matz-Soja, 2014; Kietzmann, 2017; Soto-Gutierrez et al., 2017; Ahn et al., 2019; Ko and Monga, 2019). It is impossible to build such position-specific differences in cell function with conventional static culture in a culture dish. This is because the formation of hepatic zonation requires a concentration gradient of various factors that is formed as blood flows through the sinusoidal structure, with oxygen and nutrients being consumed in the portal region of the sinusoid waste products are accumulated in the central venous region. In a conventional culture in a culture dish, changes in the medium are not location-dependent; furthermore, diffusion and convection make the entire medium uniform. In MPS, cells are usually cultured in a closed microspace, so that they are perfused with medium to maintain oxygen supply and buffering by carbonic acid. It is known that the fluid flowing through such a microspace forms an undisturbed flow, called laminar flow (Gilbert et al., 2007). In fact, such laminar flow can easily be formed even when perfusing with commercial MPS used in our laboratory (Figure 3) or reported by Taylor's laboratory (Li et al., 2018). *In vivo*, blood flowing through blood vessels is known to form laminar flow, and this is responsible for zonation in the sinusoids as blood flows through them (Mi et al., 2018). The development of zone-specific hepatocyte function in the sinusoids may be due to the concentration gradient of various factors formed by the laminar flow, but the exact mechanism of this process remains unclear. Perfusion culture with MPS is an appropriate approach for the reproduction of such an environment and the elucidation of the molecular mechanism of zonation.

## Considerations for Peripheral Technologies Involving MPS

**Figure 4:**
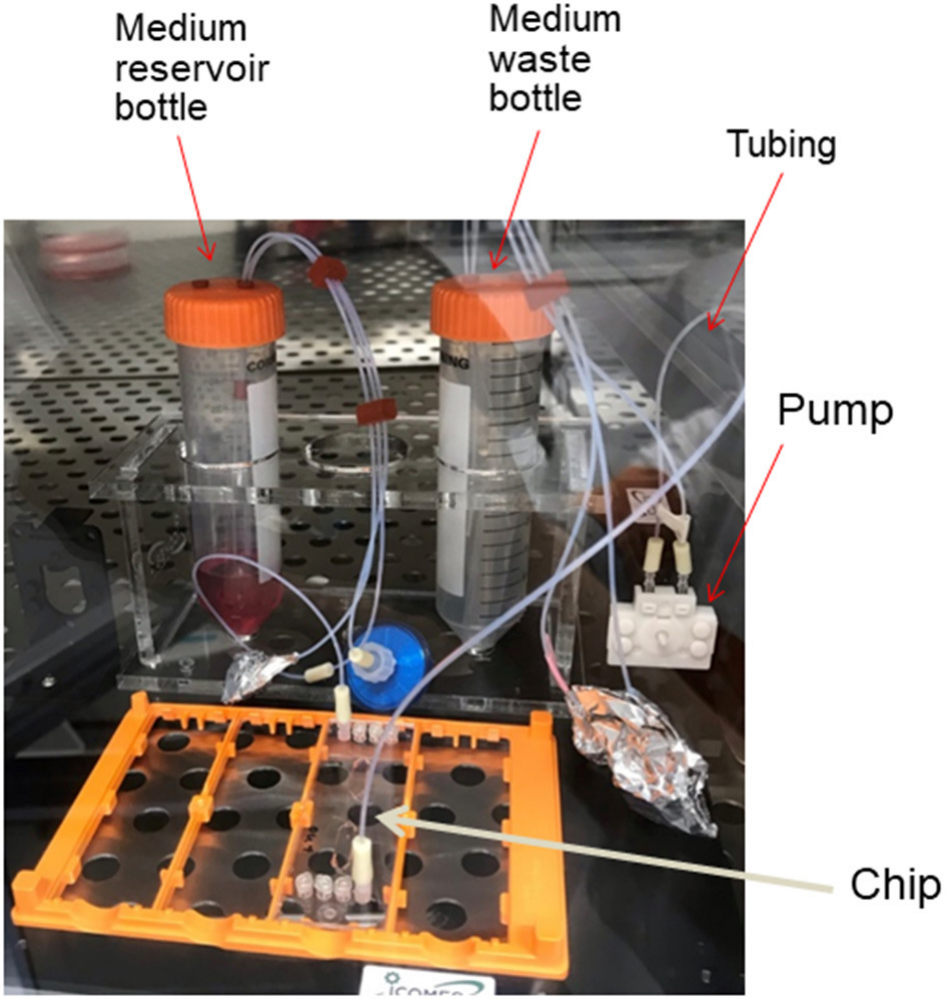
Configuration of MPS. An example of the configuration of a Barrack-type MPS assembled in our laboratory.

So far, we have discussed the characteristics of MPS from the viewpoint of the cell culture environment, but it is also important to consider them from the viewpoint of culture equipment manufacturing. Dimethylpolysiloxane (PDMS) has traditionally been widely used in the development of MPS channels because of the ease of its microfabrication process at the laboratory level. PDMS is an excellent material in terms of oxygen supply to the cell culture space, as it is porous and permeable to small molecules, including air. However, it sorbs low-molecular-weight compounds, especially low-molecular-weight lipophilic compounds, and there have been reports of cases in which the concentration of the test substance added to the culture medium is significantly reduced (van Midwoud et al., 2012). Therefore, it is important to develop a material that has less sorption and is suitable for MPS channel processing. Joint research between the Kyoto University and AGC Techno Glass reports on the development of chips made of materials with such characteristics (Sano et al., 2019). To form a flow channel, it is important to develop a technique for bonding molded parts consisting of multiple layers. When organic solvents are used for bonding, it is necessary to evaluate the cytotoxicity of the remaining solvent and its effects on the cell adhesion surface. In this respect, the bonding technology developed by USHIO (Tokyo, Japan) using a special wavelength of light has advantages in terms of cytotoxicity^16^. When conducting long-term cell culture, there are many other considerations that have already been resolved with conventional culture dishes, such as sterilization of the MPS, maintenance of sterility during the culture period, and the possibility of observing cells over time. In addition, unlike conventional culture dishes, MPS are characterized by the fact that cells are cultured in a closed space, and cells of different origins can be linked and cultured. When the culture space is closed or minute, it is necessary to supply oxygen and culture medium components required by the cells and to remove waste products. In addition, when different cells are connected and cultured, the medium needs to be transferred back and forth between the cell culture compartments. For this purpose, MPS are often capable of perfusing the culture medium. The MPS is a toolbox for this purpose, consisting of a circulation pump, reservoirs for culture medium and waste fluid, tubing to connect them, and connectors to form a single culture platform. An example of the configuration of a Barrack-type MPS in our lab is shown in Figure 4. When considering the MPS as a cell culture platform, there are points to be considered not only in the instrumentation needed to make the culture vessel, but also in the standards of the pump, the material of the tubing used for circulation, and the material of the reservoir. Discussions on these issues have begun not only in MPS (Low et al., 2020), but also in microfluid-based medical devices (Reyes et al., 2021). For example, if the entire circulation system is to be installed in a culture incubator, the pump used for circulation must be able to operate safely in a 100% humidity environment. In addition, the heat generated during operation must not alter the 37°C culture environment. Silicone tubing is sometimes used for circulation because it is easy to handle; however, silicone has the same sorption problems as PDMS, making it necessary to develop an alternative. Moreover, it is necessary to sterilize not only the culture vessel but also the entire perfusion culture system, and maintain sterility during the culture period; this requires that the material should be able to withstand sterilization processes such as gamma-ray irradiation or ethylene oxide gas sterilization. It is also important to simplify the flow path and perfusion devices. Seesaw shaker perfusion methods such as Mimetas and PD-MPS (Satoh et al., 2017), which use the pneumatic pumping method developed in the AMED-MPS project, are interesting solutions.

As mentioned above, discussions on MPS are required, not only on the cells and culture environment, but also on the culture equipment and perfusion devices as a platform; however, discussions on MPS in Europe and the United States have focused on the former and have not sufficiently discussed the equipment and devices. The AMED-MPS project has taken advantage of the participation of suppliers who produce and provide MPS, and has established a working group to study materials.

## Future of MPS

Considering its cost effectiveness as a platform, we have discussed the applications of MPS in the evaluation of drug-induced liver injury, with a view of its application in drug development. Pharmacokinetic, safety, and toxicity evaluations are common areas of interest in drug development, and it is expected that common MPS for multiple drug development can be deployed. In the United States and Europe, such discussions have been held, and reports have been published (Franzen et al., 2019; Allwardt et al., 2020; Ramadan and Zourob, 2020). Since the market size is expected increase, government-led project-based developments such as the AMED-MPS project, in which suppliers and users participate, are useful, and we hope for continued support in the future. Moreover, the strength of human *in vitro* assays such as MPS is likely to be demonstrated in the efficacy evaluation stage of drug development. However, drug efficacy evaluation cannot be handled by one type of platform, and customization of MPS is essential. Because users are usually limited to a single pharmaceutical company for each target disease, the MPS must be provided in a form that allows for small-scale, multi-species production, and the corresponding quality control. The development of this type of MPS is more appropriate for the private sector than for publicly funded projects; however, there are already several companies in Japan that are considering this type of MPS project, and we look forward to its future development as a small and flexible platform. In addition, considering the complexity of the culture equipment and the use of expensive human frozen hepatocytes, it is not easy to expand the number of users of MPS to industries other than the pharmaceutical industry. However, in the field of cosmetics and chemicals, in line with the trend of reducing animal testing, the EU has already prohibited animal testing in cosmetics development (Rogiers et al., 2020). In the US, the Environmental Protection Agency (EPA) is moving to stop funding for animal testing conducted to evaluate the safety of chemicals^17^. In line with this trend, there is an urgent need to develop experimental systems that can replace animal testing in fields other than the pharmaceutical field; MPS is one such alternative candidate. However, if we take as an example, the assessment of the human health effects of chemical substances, there is a wide variety of chemical substances to be tested. In addition, in animal experiments, the information obtained from a single oral administration test includes a wide range of biochemical data, such as those obtained from pathological specimens of multiple organs and blood samples, while *in vitro* test systems require multiple test systems for each test item. For the MPS currently under development in the AMED-MPS project, it would be important to improve its ease of handling and reduce the cost per test. As we have already seen, each MPS currently available on the market is specialized for each MPS device, so to handle MPS devices from multiple companies, it is necessary to install their respective drive systems. Technical proficiency in each drive system is also required. To reduce these financial and technical hurdles, the AMED-MPS project is working to improve operability and reduce the initial cost of introducing MPS devices by sharing the same drive system for MPS devices being developed in multiple laboratories. This approach is a characteristic of project-based development involving multiple laboratories, and we expect it to become a strength of the MPS developed in Japan.

## Author Contributions

The author confirms being the sole contributor of this work and has approved it for publication.

## Conflict of Interest

The author declares that the research was conducted in the absence of any commercial or financial relationships that could be construed as a potential conflict of interest.

## Footnotes

^1^ https://www.fda.gov/science-research/about-science-research-fda/advancing-alternative-methods-fda

^2^ https://h2020-orchid.eu/

^3^ https://euroocs.eu/

^4^ https://ncats.nih.gov/

^5^ https://www.iqmps.org/

^6^ https://iqconsortium.org/

^7^ https://www.darpa.mil/program/microphysiological-systems

^8^ https://cn-bio.com/

^9^ https://mimetas.com/app-notes

^10^ https://www.emulatebio.com/

^11^ https://nortisbio.com/

^12^ https://www.tissuse.com/en/

^13^ https://insphero.com/

^14^ https://www.microfluidic-chipshop.com/

^15^ https://www.micronit.com/

^16^ https://www.ushio.co.jp/en/technology/casestudy/101003.html

^17^ https://www.epa.gov/research/epa-new-approach-methods-efforts-reduce-use-animals-chemical-testing
